# Lactoferrin, the Moonlighting Protein of Innate Immunity

**DOI:** 10.3390/ijms242115888

**Published:** 2023-11-02

**Authors:** Antimo Cutone, Giovanni Musci, Maria Carmela Bonaccorsi di Patti

**Affiliations:** 1 Department of Biosciences and Territory, University of Molise, 86090 Pesche, Italy; antimo.cutone@unimol.it (A.C.); giovanni.musci@unimol.it (G.M.); 2 Department of Biochemical Sciences, Sapienza University of Roma, 00185 Rome, Italy

Lactoferrin (Lf), a naturally occurring glycoprotein involved in innate immunity, was first discovered in bovine milk [1] and later purified from human milk [2]. In recent years, Lf has become increasingly attractive to researchers from different fields due to its multiple functions and applications. In addition to its initially discovered antimicrobial effects [3], Lf is currently recognized as a potent multi-target nutraceutical, endowed with immunomodulatory [4], anti-inflammatory [5], anti-oxidant [3] and anti-cancer [6] properties. Of note, Lf shows a wide range of tolerability, being classified as a “generally recognized as safe” (GRAS) substance by the U.S.A. Food and Drug Administration [7] and as a dietary supplement by the European Food Safety Authority [8]. At a mechanistic level, some of the functions exerted by Lf have been related to both its iron-binding ability and its highly cationic charge, which enables it to interact with a wide repertoire of host and pathogen receptors and antigens [9,10]. However, most of its biological effects have yet to be fully appreciated and unveiled. Moreover, Lf has recently emerged as a potent carrier for the delivery of biologically active nanoparticles [11,12].

This Special Issue, entitled “Lactoferrin, the Moonlighting Protein of Innate Immunity”, consists of five articles selected for publication.

Bukowska-Ośko and colleagues reviewed the most recent studies about Lf’s ability to defend the host from DNA damage induced by chemical and biological agents [13]. Such changes in DNA structure and sequence can cause premature aging, cell degeneration and death, thus leading to severe tissue and organ failure [14]. Several diseases, including cancer, have been associated with this process, so recent research has focused on promising compounds that can counteract/reverse these effects [15,16]. In this scenario, natural products, which have evolved to prevent disease and repair damaged genetic material, are emerging as safe, well-tolerated and adjuvant substances for the maintenance of body homeostasis, including genome integrity. The review by Bukowska-Ośko et al. presents the new and innovative role of Lf in the protection of human genetic material against internal and external damage, described via cell cycle modulation mechanisms at all its levels and repair mechanisms [13].

As an ancestral guardian, the primary defense activities of Lf against pathogens and their sequelae have been dissected in different studies in the present Special Issue. Li and colleagues have investigated the beneficial effect of Lf administration in a model of lipopolysaccharide (LPS)-induced intestinal immune barrier damage [17]. The innate immune barrier of the intestinal tract plays a crucial role in human health, especially in infants and young children who have an immature immune system [18]. First, pharmacokinetic analyses of Lf in mice intestinal tissues, stomach tissues and blood revealed the higher efficacy of oral gavage in improving Lf bioavailability with respect to intraperitoneal injection. In addition, through in vitro and in vivo models of infantile intestinal immune barrier damage, Lf was demonstrated to significantly increase the survival rate of LPS-induced primary intestinal epithelial cells and downregulate the expression of inflammatory cytokines, such as interleukin (IL)-1β, IL-6, tumor necrosis factor (TNF)-α and interferon (IFN)-γ, in both primary intestinal epithelial cells and blood of mice. Interestingly, the beneficial effects of Lf treatment were correlated with the rate of iron saturation of the glycoprotein, with higher efficacy for Apo-Lf than for the Holo form. Next, the related immune molecular mechanism, investigated using RNA-seq assay, revealed that ELAVL1 (embryonic lethal abnormal vision Drosophila 1) is one of the key genes regulated via Lf treatment, and the ELAVL1/PI3K/NF-κB pathway was shown to participate in Lf-mediated protection of infantile intestinal immune barrier damage. In addition, the ratio of blood CD4+/CD8+ T cells was significantly higher in the Lf-treated mice than in the control mice. Lactoferrin was therefore proven to alleviate LPS-induced intestinal immune barrier damage in young mice through the regulation of ELAVL1-related immune signaling pathways, which would expand the current knowledge of the functions of bioactive proteins in food within different levels of research, as well as benefit preclinical and clinical research in the long run [17].

The antiviral activity of Lf has been widely recognized [19,20], and the recent spread of Severe Acute Respiratory Syndrome coronavirus 2 (SARS-CoV-2), the causative agent of coronavirus disease (COVID)-19, has renewed the interest in this glycoprotein in this field [21]. Several in vitro [22,23] and clinical studies [24,25] have pointed out the possible employment of Lf, especially the bovine form, as an adjuvant of standard-of-care therapy for COVID-19 patients [26]. In this framework, the study by Piacentini et al. was aimed at unraveling the putative interactions between human Lf (hLf), SARS-CoV-2 receptor-binding domain (RBD) and human angiotensin-converting enzyme 2 (ACE2) receptor, in order to provide a molecular basis for the reported preventive effect of Lf against CoV-2 infection [27]. Specifically, kinetic and thermodynamic parameters of the pairwise interactions between the three proteins, carried out via both biolayer interferometry and latex nanoparticle-enhanced turbidimetry, revealed that hLf was able to bind the ACE2 receptor ectodomain with significantly high affinity, while no binding to the RBD was observed up to the maximum “physiological” concentration range of Lf. Indeed, above a concentration of 1 µM, hLf appeared to directly interfere with RBD–ACE2 binding, bringing about a measurable, up to 300-fold increase of the K_D_ value relative to RBD–ACE2 complex formation [27].

The protective role of Lf against pathogenic processes related to the expression of the sole viral proteins was also reported in the study by Ianiro and colleagues, where iron saturation rate was found to drive bLf effects on oxidative stress and neurotoxicity induced via HIV-1 Tat [28]. Despite tremendous advances in combined antiretroviral therapy, the standard therapy for HIV-1 infection, HIV^+^ patients can experience HIV-associated neurocognitive disorders [29], mainly due to the chronic basal expression of HIV-1 non-structural proteins, such as Tat, which can trigger neurotoxic effects, related to both oxidative stress induction and excitotoxicity [30,31]. BLf was found to reduce oxidative stress and oxidative damage in astrocytes expressing the Tat protein of HIV-1. Additionally, Lf restored the normal function of iron uptake in cells expressing the viral protein. However, the effects of the glycoprotein varied depending on its iron saturation, as the Holo form was found to exacerbate Tat-induced excitotoxicity via up-regulation of System Xc^−^, thus highlighting the utmost importance of considering Lf iron saturation when applied to clinical therapies, as it can dramatically influence the success or failure of the treatment itself [28]. 

In this framework, the role of iron in driving Lf activities was also remarked in the study by Lian and colleagues in which, as a natural defense agent in ocular wellness [32,33], Lf was explored as a promising tool in the treatment of myopia in a murine model [34]. Mice were given different forms of Lf, such as native Lf, Holo Lf and digested Lf, for 3 weeks before inducing myopia with minus lenses. The results showed that mice given digested Lf or Holo-Lf had a less elongated axial length and thinned choroid compared with those given native-Lf. Gene expression analysis also showed that the groups given Lf, both in native and Holo forms, and its derivatives had lower levels of certain cytokines, such as IL-8 and TNF-α, and growth factors, such as vascular endothelial growth factor (VEGF), associated with myopia, compared with the PBS-treated group. The authors conclude that oral administration of digested Lf can suppress myopia more effectively than the administration of native-Lf. Additionally, Holo-Lf can suppress choroidal thinning more effectively than native-Lf with low saturation, again confirming the primary role of the metal in Lf biological functions. 

This Special Issue was effective in bringing together new and promising studies in the lactoferrin field, emphasizing the potential application of this natural product in current and future research as an effective alternative or complement to standard therapy.
